# Validation of the WHOQOL-Bref: psychometric properties and normative data for the Norwegian general population

**DOI:** 10.1186/s12955-020-01656-x

**Published:** 2021-01-07

**Authors:** Mary H. Kalfoss, Randi J. Reidunsdatter, Christian A. Klöckner, Marianne Nilsen

**Affiliations:** 1 Faculty of Health Studies, VID Specialized University, Campus Diakonova, Pb 184, Videren, 0319 Oslo, Norway; 2 Department of Circulation and Medical Imaging, Norwegian University of Science and Technology, Trondheim, Norway; 3 Department of Psychology, Norwegian University of Science and Technology, Trondheim, Norway; 4 Department of Social Work, Norwegian University of Science and Technology, Trondheim, Norway

**Keywords:** WHOQOL-bref, Validity, Reliability, Psychometric properties, Invariance, Normative data

## Abstract

**Background:**

The World Health Organization’s Quality of Life Questionnaire (WHOQOL-Bref) is a frequently used instrument to assess the quality of life in both healthy and ill populations. Inquiries of the psychometric properties of the WHOQOL-Bref report that the validity and reliability is generally satisfactory. However, some studies fail to support a four-factor dimensionality; others report poor reliability of the social and environmental domain; and there may be some challenges of supporting construct validity across age. This paper evaluates the psychometric properties of the Norwegian WHOQOL-Bref and extends previous research by testing for measurement invariance across age, gender and education level. In addition, we provide updated normative data for the Norwegian population.

**Methods:**

We selected a random sample of the Norwegian population (*n* = 654) aged 18–75 years. Participants filled out the WHOQOL-Bref, the Utrecht Work Engagement Scale and various sociodemographic variables.

**Results:**

We found an acceptable convergent and discriminate validity and internal consistency of the physical, psychological and environmental domains, but a marginal reliability was found for the social domain. The factor loadings were invariant across gender, education and age. Some items had low factor loadings and explained variance, and the model fit for the age group 60–75 years were less satisfactory.

**Conclusions:**

The original four-factor dimensionality of the WHOQOL-Bref displayed a better fit to the data compared to the one-factor solution and is recommended for use in the Norwegian population. The WHOQOL-Bref is suitable to use across gender, education and age, but for assessment in the oldest age group, the WHOQOL-Old module could be a good supplementary, but further studies are needed.

## Introduction

Recent years have witnessed considerable interest in quality of life (QoL) research which spans multiple disciplines [1–3]. This international interest has been impacted by people living longer, the increase in chronic conditions and rising costs of healthcare delivery [4–7]. Health professionals and researchers also agree that health services, policy making, and the efficacy of treatment interventions should be evaluated by its impact on QoL [8, 9]. These developments have resulted in a proliferation of assessment instruments [6, 10–12]. The World Health Organization’s Quality of Life Questionnaire (WHOQOL-Bref) is one of the most known generic questionnaires for the assessment of QoL in both healthy and ill populations [3, 10, 13, 14]. Over 20 years, a WHOQOL-Bref manual has facilitated around 100 culturally adapted translations of this instrument globally and completed by over 60,000 adults from both healthy and diseased populations [15]. Validation of measurement instruments, the WHOQOL-Bref included, is an ongoing process—and accumulated evidence of validity is needed if any inferences and interpretations of instrument scores are to be supported [16]. Although inquiries of the psychometric properties of the WHOQOL-Bref report that the validity and reliability of the scale is generally satisfactory [10, 13, 14], some inquiries fail to support the theoretical four factor dimensionality of the WHOQOL-Bref without adding modifications to the instrument—and sometimes a poor reliability of the social and environmental domain is evident [14, 17–20].

Furthermore, support of construct validity in terms of measurement invariance is reported by some studies [21, 22], but not others [23], and one study reported measurement invariance across gender, but not across age [2].

Finally, normative cross-cultural data are also relatively scarce given the worldwide use of the instrument [24–26]. The WHOQOL-Bref was translated for use in Norway according to WHO international guidelines [27], but no population norms from Norway have been provided in over 15 years [28].

Thus, in the current inquiry, we evaluate the psychometric properties of the Norwegian WHOQOL-Bref, taking advantage of a Norwegian general population study. We aim to replicate previous investigations of psychometric properties, but also extend existing research by testing for measurement invariance across age, gender, and education level, and lastly provide updated normative data for the Norwegian population.

### Psychometric qualities of the WHOQOL-Bref

Construct validity refers to the ongoing process of examining the theoretical relationship between items and to the hypothesized scale [29]. Despite the frequent use of the WHOQOL-Bref and evidence for its psychometric soundness, questions remain about whether data are well presented by the theorized four-factor structure, and whether the WHOQOL-Bref is measuring the same structure in different populations.

The results of several inquiries support an appropriate fit of a four-factor structure of Qol in general populations [3, 25, 26, 30] and in disease populations [18–20]. However, rescoring or omitting items to conform to acceptable fit indices of the four-factor model is reported [31, 32]—and although items are found to correlate most strongly with their theoretically intended domain, the items may correlate highly across other domains as well [14, 33]. Indeed, reports on modified versions of the four-factor structure of the WHOQOL-Bref is quite common [32], which was also found in the earlier Norwegian population study by Hanestad and colleagues [28]. High correlations between items across domains have also lead to questions whether the WHOQOL-Bref is best represented by one domain of overall QoL [33]. Good fit of data to a one-factor structure is also supported by others [30, 34].

Testing for factorial invariance of a measurement instrument is an important step in the evaluation of the scales construct validity [35]. When an instrument operates equally, and the underlying constructs have the same theoretical structure across different groups, evidence of factorial invariance is strengthened [35]. In a Taiwanese national survey, evidence of measurement invariance of the WHOQOL-Bref was supported, after controlling for age and gender among healthy and disease populations, between disease and matched healthy groups and across disease groups [21]. One study, among 1972 undergraduates from nine Spanish-speaking countries, found evidence of factorial invariance of the WHOQOL-Bref across countries, even though the initial testing yielded a poor fit to the original 4 factor theoretical model [26]. The final model showed a structure which was a different and more complex configuration from that of the original. The social domain, originally tapped by items 20 and 21, was in this new factor structure tapped by items 10, 11, 12, 19, 20, 23, 24 and 25. Other findings are not as supportive of invariance across nations; Theuns and colleagues [23] explored whether the scale measured the same construct across Belgium and Iran and found that eleven out of 24 items had invariant factor loadings and thresholds, mainly in the physical and psychological domains.

Perera, Izadikhah, O'Connor and McIlveen [2] explored competing latent structures in a general Australian population and investigated the retained model across gender and age. Their findings supported a two-factor solution with measurement and structural invariance across gender. A curvilinear relationship between age and the QoL domains were evident—thus the QoL dimensions might not be comparable across younger and older individuals.

Based on the above, measurement invariance of the WHOQOL-Bref is supported across some countries, healthy versus disease populations, and across gender. Invariance across age is, however, less certain.

### Reliability and scaling qualities

Although most studies support the psychometric fitness of the physical and psychological health domains, several studies have reported low internal consistency of the social domain [17, 18, 36], as was found in the older Norwegian study [28]. The item focused on safety is also shown to have low internal consistency with the environmental domain [33].

Ceiling effects is a well-known problem in QoL research and indicates that items/scales have poor discrimination and thus impaired sensitivity and responsiveness [37]. In a comprehensive study with WHOQOL-Bref data from 23 countries, results indicated that the 5 items—cognitive ability, body image, information, personal relationships and access to health services—had marginally skewed distributions with few responses (< 10%) at the lower ends of the scale [10].

### Study aims

Based on data from a random sample of the Norwegian population, the primary aim was to examine construct validity and reliability of the Norwegian WHOQOL-Bref, addressed by the following research questions:

1. Does the original four-factors model of the WHOQOL-Bref have a better fit than the one-factor model to the general Norwegian population data?
2. Does the four-factors model of the WHOQOL-Bref reveal satisfactory construct validity in terms of dimensionality, convergent and discriminant validity, and reliability (internal consistency, floor-ceiling) in the general Norwegian population?
3. Are the underlying dimensions of the WHOQOL-Bref stable (invariant) across gender, age and education?

A secondary aim was to generate up-to date Norwegian normative data for the WHOQOL-Bref.

## Methods

### Procedure and sample

A random sample of 3000 individuals was selected from the Norwegian population in 2009 in two steps: first, a sample of 2500 individuals aged 18–75 years was drawn, followed by an additional 500 individuals aged 60–75 years, as we expected lower response rates for elderly people. The samples were drawn from individuals listed in the Norwegian National Population Register. A questionnaire was sent by mail to the 3000 persons who were selected; n = 57 were returned due to unknown addresses or death; 29 persons declined participation for unknown reasons; 2260 persons did not respond; and 654 (22%) chose to participate in the study by returning the questionnaire by prepaid post. A reminder was sent four weeks after the first mailing.

### Measures

The WHOQOL-Bref contains one item from each of the 24 facets from the WHOQOL-100, as well as two single items on overall QoL and health satisfaction [38]. The 26 items produce 4 domains related to QoL; physical (health), psychological, social relationships and environmental and an overall QoL and health satisfaction facet. Each item is measured from 1 to 5 on a Likert scale, with varying scale response anchors, where higher values represent higher QoL. One example of item is “How much do you enjoy life?”, rated on the following response options (1) not at all, (2) a little, (3) a moderate amount, (4) very much, and (5) an extreme amount. The domain scores were calculated by multiplying the mean score of each domain by four according to WHOQOL-Bref scoring manual. The time span covers the past 2 weeks. The two single items of the WHOQOL-Bref “How would you rate your quality of life” and “How satisfied are you with your health” were used to examine the convergent validity of the WHOQOL-Bref. The Norwegian version of this scale was translated according to the WHO translation protocol [27].

The Utrecht Work Engagement Scale short version (UWES-9) was applied to explore the discriminant validity of the WHOQOL-Bref—by examining that WHOQOL-Bref was not to highly correlated with instruments designed to measure other concepts. Work engagement is defined as a positive, fulfilling state of mind related to work, and is supposed to be moderately correlated to the four domains of Qol and with overall Qol. The 9 items covering the domains of vigor, dedication, and absorption were rated on response options ranging from (1) “never” to (7) “always (every day)”, and then summed to form a single score of work engagement. The psychometric properties of the UWES-9 is found satisfactorily [39]. Cronbach’s alpha of the UWES-9 in the current study was 0.94.

### Data analysis

Data were screened and analyzed using SPSS version 24.0 [40] and Mplus version 8.0 [41]. All 26 items of the WHOQOL-Bref were screened for ceiling and floor effects by examining the skewness and kurtosis for each item.

Confirmatory Factor Analysis (CFA) with the mean- and variance-adjusted weighted least squares (WLSMV) estimator was used to model the original hypothesized four constructs of the WHOQOL-Bref. The five-point Likert answering scales were treated as ordered categorical variables in the CFA analysis. Two statistical measures were used to assess the fit of the CFA-models; the Root Mean Square Error of Approximation (RMSEA) and the Residual Mean Squared Error of Approximation (SRMR) [42]. The cutoff criteria for determining good model fit was following Hu and Bentlers [42] recommendations of a RMSEA < 0.06 and SRMR < 0.08. Adjusted Chi square difference testing in Mplus for WLSMV estimator using the DIFFTEST method was used to test for significant differences between the nested models. In the interpretation of the Chi square result we used both the significance level and the Chi square to degrees of freedom ratio (with a cut-off of a ratio of 2) as Chi square tests have a tendency to be oversensitive in larger samples as outlined in Byrne [43].

Multiple-group CFA models were used to evaluate the measurement invariance of the identified WHOQOL-Bref structure across gender (men vs. women), age (younger [18–39] vs. middle aged [40–59] vs. older age [60–75]), and education (primary/secondary school vs. high school vs. college/university). To establish measurement invariance, we evaluated three different models for each group (gender, age, education) for a significant decrease in model fit assuming stricter versions of measurement invariance (Model 1–3, see below). In the multiple-group CFA analyses, category 1 and 2 on the five-point Likert scale was collapsed due to few or missing responses in category 1 in some items in some subgroups. In Model 1 (configural invariance), all parameters were free to vary across groups, but the structure of the models were constant across subgroups. In Model 2 (metric invariance) the factor loadings were constrained to be equal across groups, residual variances were fixed at one in one group and free in the other groups, and factor means were fixed at zero in one group and free in the other groups. The first threshold of each item was held equal across groups. The second threshold of the item that is used to set the metric of the factor were held equal across groups. Factor variances were free across groups [41]. In Model 3 (scalar invariance) both factor loading and thresholds were held equal across groups. Metric invariance (invariant factor loadings) was established between the groups if Model 2 did not have a significantly poorer fit than Model 1, and scalar invariance (same constructs are measured on similar scale) was established if Model 3 did not have a significantly poorer fit compared to Model 2. Missing data was handled using the procedure of Full Information Maximum Likelihood (FIML) that allows for estimation of a model using all available information. Missing data for the WHOQOL-Bref single items ranged from 1.8 to 5.2%.

Convergent and discriminant validity were evaluated by examining their relationship between the four domains of WHOQOL-Bref and UWES-9, overall QoL and satisfaction with health using Pearson’s product-moment correlation coefficient analysis.

To provide population norms, the mean and standard deviation was calculated on weight adjusted data. An adjustment weight was added to each participant based on the population distribution of gender (men, women), age (18–39; 40–59; 60–75 years), and education (primary/secondary school, high school, college/university). The weighting efficiency was 83.09%, and the range of applied weights were 0.34–4.82. Internal consistency reliability was examined by calculating Cronbach’s alpha for all domains. Due to the unidimensional hypothesis, Cronbach’s alpha was also calculated for the entire scale.

## Results

### Demographics

The sociodemographic characteristics of age, gender and education are displayed in Table 1. Compared to the population, the sample consisted of fewer participants from the younger (18–39 years) and a higher proportion og the older (60–75 years) age group, and proportionally more women and people with higher levels of education.

**Table 1:** Sociodemographic characteristics; mean, SD, n (%) of age, gender, education and marital status among participants and non-responders

Mean age (SD)	Participants	Non-responders	Population
	n = 654	n = 2346	n = 3,827,770
	50.3 (16.2)	47.8 (17.1)	–
	n (%)	n (%)	n (%)
Age groups			
18–39	188 (30)	846 (36)	1,529,499 (40)
40–59	218 (35)	736 (31)	1,296,027 (34)
60–75	226 (36)	765 (33)	1,002,244 (26)
Gender			
Women	345 (54)	1162 (50)	1,930,370 (50)
Men	299 (46)	1185 (51)	1,897,400 (50)
Educational level			
Primary/Secondary school	81 (13)	–	1,116,735 (29)
High school	273 (43)	–	1,597,491 (42)
College/University	284 (45)	–	1,005,365 (26)
Marital status			
Married or partnered	484 (74%)	–	–
Single	78 (12%)	–	–
Separated/divorced/widowed	78 (12%)	–	–

No data on level of education or marital status for non-responders is available. No data on mean age and marital status are provided for the population data

The study participants were significantly older than non-responders, mean age (SD) = 50 (16.2) versus 48 (17.1) years, *p* < 0.001, but no significant gender difference was observed (*p* = 0.069).

Twenty-five percent were senior citizens, and 63% were employed workers. Eighty-three percent rated their overall QoL (WHOQOL-Bref single item on QoL) to be good/very good; and 74% were satisfied/very satisfied with their health (WHOQOL-Bref single item on health). Work engagement was high among participants with a mean of 5.9 (SD = 1.2) on a scale from 1 to 7, where higher numbers represent more work engagement.

### Scaling qualities

**Fig. 1:**
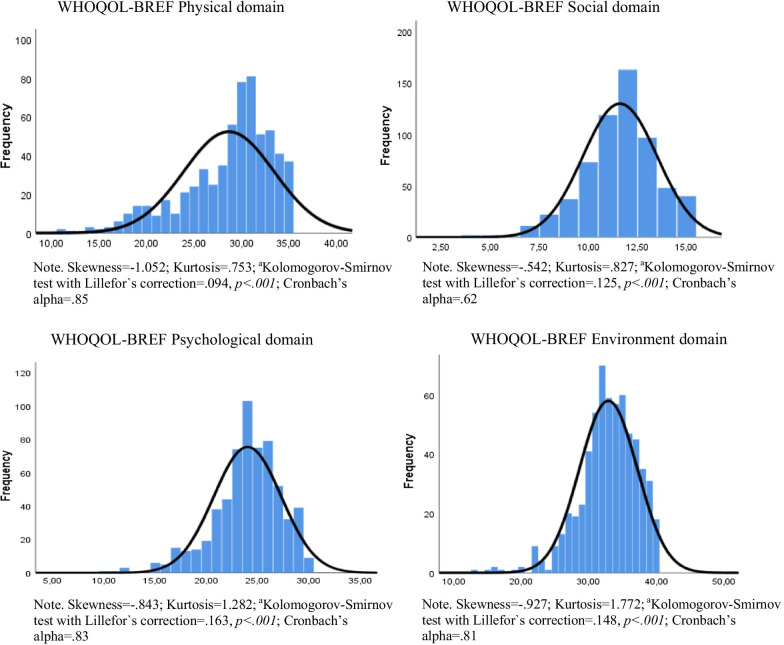
Frequency distribution of the four domains of the WHOQOL-BREF

All 26 items in the WHOQOL-Bref were skewed left, indicating ceiling effects for all items. Both single items and the four domains showed non-normal distributions (Fig. 1).

### Construct validity

#### Dimensionality

The results of the CFA for the entire WHOQOL-Bref are presented in Table 2. All fit indices regarding the one-dimensional structure suggest that this model does not fit the data well. Compared to the one-factor model, the original four-factor model significantly improved the fit of the data, *X*^2^ (6, *N* = 644) = 465.764, *p* < 0.001. However, the original 4-factor model did not yield a good fit according to the fit indices. All the 26 items loaded significantly on their respective latent factors, and the loadings ranged between 0.513 and 0.933. One item had R^2^ < 0.30 (item 4 on the physical subscale), and a few others had R^2^ < 0.40 (Item 3 on the physical subscale; item 11 on the psychological subscale; item 9, 12, 24 and 25 on the environmental subscale; and item 21 on the social subscale). All subscales showed high positive correlations with each other, ranging from 0.608 to 0.839.

**Table 2:** Results of confirmatory factor analysis of the WHOQOL-Bref

Model alteration	RMSEA	SRMR	χ^2^ (df)
Model 1: One factor	0.113	0.077	2326.360 (252)***
Model 2: Four factors	0.087	0.060	1436.571 (246)***

RMSEA, root mean squared error of approximation; SRMR, Standardized Root Mean Square Residual

^***^p < 0.001

#### Model modifications

The hypothesized four-factor model did not yield an adequate model fit. Subsequent CFA’s were therefore carried out to explore the sources of misfit with a goal of establishing a substantively viable model. We split our sample randomly in two halves (*n* = 321 and *n* = 331) to avoid the possibility of capitalizing on sample-specific variance that may spuriously inflate model fit. The modifications of a four-factor-model were explored in one half of the sample (Sample A), and cross-validated in the other half (Sample B). The four-factor-solution in Sample A was not adequate according to fit indices (RMSEA = 0.078, SRMR = 0.061). In search for model misspecification, we examined the Modification Indices (MI), successively addressing parameters with the largest MI and Expected Parameter Change (EPC), one at a time. We allowed the measurement error of item 5 and 6 (MI = 104.636; EPC = 0.298), item 3 and 4 (MI = 64.150; EPC = 0.38), and item 24 and 25 (MI = 56.050; EPC = 0.313) to be correlated. It is likely that each of the three pairs of items had something in common other than the latent construct. Item 5 and 6 asked about meaning and satisfaction in life; item 3 and 4 were about pain and medical treatment; and item 24 and 25 asked about access to health services and transport. Each pair of items were from the same domain. All factor loadings were > 0.404 in the modified measurement model across the three samples (Table 3). All *R*^*2*^ were all ≥ 0.20, but a few items were in the lower range of *R*^*2*^ across the three samples (item 3, 0.286–0.349; item 4, 0.200–0.227; item 24, 0.282–0.285; item 25, 0.278–0.331). The modified total model had acceptable fit to the data across all indices (Table 4). The RMSEA of sample B was 0.075, indicating a poorer fit compared to sample A and the total sample. All QOL domains were all positively correlated in all three samples (Table 5).

**Table 3:** Standardized factor loadings of the modified four-dimensional measurement model for sample A, B, and the total sample

Scale	Item	Sample A	Sample B	Total sample
Physical	Item 3	.535	.591	.564
	Item 4	.447	.476	.460
	Item 10	.847	.812	.829
	Item 15	.746	.840	.794
	Item 16	.656	.628	.643
	Item 17	.923	.941	.933
	Item 18	.881	.892	.885
Psychological	Item 5	.771	.758	.762
	Item 6	.773	.763	.766
	Item 7	.735	.597	.667
	Item 11	.647	.555	.598
	Item 19	.896	.859	.877
	Item 26	.659	.705	.679
Social	Item 20	.866	.720	.792
	Item 21	.591	.611	.605
	Item 22	.710	.681	.697
Environmental	Item 8	.802	.760	.781
	Item 9	.628	.607	.619
	Item 12	.648	.606	.627
	Item 13	.652	.746	.699
	Item 14	.671	.713	.691
	Item 23	.715	.639	.677
	Item 24	.531	.543	.535
	Item 25	.527	.576	.555

All loadings were statistically significant at *p* < .001

**Table 4:** Fit indices of modified models of sample A, B and total sample

Sample	RMSEA	SRMR	χ^2^ (df)
Sample A	0.059	0.052	513.157 (243)***
Sample B	0.075	0.061	687.528 (243)***
Total sample	0.067	0.051	955.090 (243)***

RMSEA, Root Mean Squared Error of Approximation; SRMR, Standardized Root Mean Square Residual

^***^p < 0.001

**Table 5:** Correlations among latent domains of the modified version of QOL-BREF

Subscales	Physical	Psychological	Social	Environmental
Sample A (*n* = *318*)				
Physical	–			
Psychological	.83***	–		
Social	.61***	.85***	–	
Environmental	.80***	.86***	.68***	–
Sample B (*n* = *326*)				
Physical	–			
Psycological	.80***	–		
Social	.63***	.95***	–	
Environmental	.82***	.80***	.73***	–
Total sample (*n* = *644*)				
Physical	–			
Psychological	.82***	–		
Social	.61***	.89***	–	
Environmental	.81***	.83***	.70***	–

^*^p < 0.05; **p < 0.01; ***p < 0.001

#### Factorial invariance across gender, age, and education for the modified WHOQOL-Bref

In the modified WHOQOL-Bref, we tested for measurement invariance across gender, age and education (Table 6). Initially, the CFA models were estimated separately for each group (i.e. gender etc.), and in a multiple group CFA with no constraints imposed (Model 1). Evidence of configural invariance was supported across gender and education—as the separate models and Model 1 all had a marginal, but acceptable goodness of fit. The separate models ran for each age group showed an acceptable fit for ages 18–39, marginally acceptable fit for ages 40–59, but for persons 60–75 years of age the model fit was poorer with a RMSEA = 0.07. Model 1 for age showed acceptable fit to the data.

**Table 6:** Model fit and nested model comparisons for multiple group CFA analyses

Multiple group CFA	Model fit indices			Nested model comparison		*P*-value	χ^2^/df
	χ^2^ (df)	RMSEA	SRMR	Comparison	χ^2^ (df) test for difference		
Gender							
Women (*n* = *344*)	632.18 (243)	0.068	0.062				
Men (*n* = *297*)	521.71 (243)	0.062	0.058				
Model 1 (all parameters free)	1155.42 (486)	0.066	0.060				
Model 2 (constrained factor loadings)	1151.52 (506)	0.063	0.060	Model 2 versus 1	31.679 (20)	.047	1.58
Model 3 (constrained factor loadings and thresholds)	1185.97 (550)	0.060	0.061	Model 3 versus 2	71.99 (44)	.049	1.64
Age							
18–39 (*n* = *187*)	325.59 (243)	0.043	0.059				
40–59 (*n* = *218*)	467.48 (243)	0.065	0.065				
60–75 (*n* = *224*)	511.33 (243)	0.070	0.066				
Model 1 (all parameters free)	1293.64 (729)	0.061	0.063				
Model 2 (constrained factor loadings)	1314.23 (769)	0.058	0.064	Model 2 versus 1	59.82 (40)	.023	1.50
Model 3 (constrained factor loadings and thresholds)	1487.83 (857)	0.059	0.066	Model 3 versus 2	231.95 (88)	.000	2.64
Education							
No post-secondary Education (*n* = *351*)	620.65 (243)	0.067	0.060				
Post secondary Education (*n* = *284*)	499.06 (243)	0.061	0.060				
Model 1 (all parameters free)	1117.94 (486)	0.064	0.060				
Model 2 (constrained factor loadings)	1113.24 (506)	0.061	0.060	Model 2 versus 1	32.08 (20)	.043	1.60
Model 3 (constrained factor loadings and thresholds)	1154.15 (550)	0.059	0.061	Model 3 versus 2	76.82 (44)	.002	1.75

In model 2, the factor loadings were constrained to be equal across gender, age and education. According to the Chi square to degrees of freedom ratio, metric invariance was supported for all groups. In model 3 both the factor loadings and thresholds were constrained to be equal. The results showed that scalar invariance was supported for gender and education, but not for age in which the Chi square to degrees of freedom ratio was larger than 2.

#### Convergent and discriminant validity

Table 7 presents correlations between the modified version of the WHOQOL-Bref and indicators of validity. The four domains of WHOQOL-Bref were all positively correlated with work engagement, and with overall quality of life and satisfaction with health.

**Table 7:** Correlations among domains of the modified version of QOL-BREF, Work engagement (UWES-9), and overall quality of life and satisfaction with health

	QOL-Physical	QOL-Psychological	QOL-Social	QOL-Environmental
Work engagement	0.26**	0.37**	0.30**	0.30**
Overall quality of life	0.68**	0.63**	0.46**	0.52**
Overall satisfaction with health	0.73**	0.55**	0.40**	0.49**

^*^p < 0.05; **p < 0.01; ***p < 0.001

### Normative data and internal consistency reliability

The normative data of the WHOQOL-Bref for the total sample, for men and women and for the three age groups (youngest, medium, oldest) is presented in Table 8. Cronbach`s alpha was 0.85, 0.83, 0.62, and 0.81, respectively, for the physical, psychological, social, and environmental domains, and 0.92 for the total scale. The level of internal consistency was acceptable to good, although the social domain was marginally acceptable.

**Table 8:** Weighted normative data on the WHOQOL-Bref domains for total sample and by gender, age and education

Domains (number of items)	Total sample (n = 615–626)	Gender		Age			Education		
		Men (n = 287–293)	Women (n = 324–333)	18–39 years (n = 179–187)	40–59 years (n = 209–214)	60–75 years (n = 208–213)	Primary/Secondary school (n = 70–74)	High school (n = 260–267)	College/University (n = 276–282)
	Mean (SD) 95% CI	Mean (SD) 95% CI	Mean (SD) 95% CI	Mean (SD) 95% CI	Mean (SD) 95% CI	Mean (SD) 95% CI	Mean (SD) 95% CI	Mean (SD) 95% CI	Mean (SD) 95% CI
Physical (7)	16.46 (2.62) 16.26–16.66	16.69 (2.46) 16.43–16.95	16.18 (2.78) 15.86–16.50	17.06 (2.02) 16.81–17.31	16.22 (2.84) 15.88–16.56	15.74 (2.98) 15.20–16.28	15.87 (2.76) 15.42–16.32	16.37 (2.67) 16.06–16.69	16.94 (2.39) 16.63–17.25
Psychological (6)	15.93 (2.16) 15.76–16.10	16.10 (2.11) 15.88–16.33	15.73 (2.21) 15.47–15.98	15.92 (2.05) 15.66–16.17	15.93 (2.22) 15.66–16.20	15.94 (2.29) 15.53–16.36	15.39 (2.20) 15.02–15.75	15.91 (2.19) 1565–16.17	16.32 (2.04) 16.05–16.59
Social (3)	15.35 (2.60) 15.15–15.55	15.18 (2.56) 14.90–15.45	15.53 (2.63) 15.23–15.84	15.48 (2.70) 15.14–15.83	15.33 (2.57) 15.02–15.63	15.17 (2.43) 14.72–15.61	14.41 (2.78) 13.96–14.86	15.58 (2.59) 15.27–15.90	15.69 (2.33) 15.38–15.99
Environmental (8)	16.31 (2.23) 16.14–16.49	16.33 (2.27) 16.09–16.57	16.29 (2.18) 16.05–16.54	16.29 (2.22) 16.01–16.56	16.19 (2.28) 15.92–16.46	16.60 (2.13) 16.22–16.99	15.90 (2.75) 15.46–16.35	16.10 (2.07) 15.85–16.34	16.86 (1.92) 16.61–17.12

The n for each group of gender, age and education varies across domains. The minimum and maximum is reported in the table

## Discussion

The present study was centrally concerned with examining the construct validity of the Norwegian WHOQOL-Bref, and secondary with generating new normative data for this frequently used instrument. By means of data from a random sample from the Norwegian population we tested the complete factorial invariance of item responses across gender, education and age. The results of the study demonstrate acceptable validity and internal consistency (reliability) of the scale, however, the social domain demonstrated marginal reliability. Evidence was obtained that the WHOQOL-Bref was invariant across gender and education. However, scalar invariance could not be established for age. The model fit was slightly poorer for the older age group (60–75 years) compared to the younger groups.

The current study found that the hypothesized four-factor model did not yield an adequate model fit. Subsequent CFA’s were therefore carried out to explore the sources of misfit. The current investigation is in line with several inquiries that report on a poor fit of the original four-factor model [31–33, 44]. The same items are reported as problematic (i.e. low factor loadings, high error correlation, cross-loadings). Xia and colleagues [44] reported that a correlation between the items “enjoy life” and “meaningful life” would improve the fit of their model, similar to our findings. Furthermore, several studies report on ceiling effects for some items (24 “access to health services”, 25 “satisfaction with transport”, 4 “medical treatment”, 20 “personal relationships”) [14, 38]. In our study some of these same items were allowed to covary with each other or some other item (3 “physical pain” and 4 “medical treatment”; 5 “enjoy life” and 6 “meaningful life”; and 24 “access to health services” and 25 “satisfaction with transport”). Shared error variance and ceiling effects may both be the result of some common factor—other than the hypothesized latent domain—explaining variation in the data, thus representing a serious threat to the validity of the instrument. Items with high loadings on more than one domain are found to be more complex; for example item 8 (“safety in daily life”) is shown to have strong loadings to both the environmental domain and the psychological domain [33]. Likewise, item 8 and item 10 (“energy”) are both more strongly associated with the psychological domain than their intended domains [10]. When items display high loadings across several domains, this may indicate that Qol is better represented by one dimension. In diseased populations—in patients with coronary artery disease, and other populations with physical disorders and mental problems—only the one-factor solution had acceptable fit to the data [33, 34]. We might suppose that these groups of patients have a more holistic perception of QoL. That is, it has been suggested from a conceptual standpoint, it is conceivable that people possess a holistic sense of their functioning in addition to more differentiated subjective evaluations of domain-specific health and wellness. Consequently, some people may be informed by their cross-domain experiences in addition to a more differentiated subjective evaluation of specific domains which may be more context dependent [2]. However, the one-dimensional factor structure was not supported in our general population sample.

Despite that we found a slightly dissatisfactory four-factor solution to the original WHOQOL-Bref, a few modification (i.e. adding correlations between error variances of some items) resulted in a good fit to the four-factor model.

Although the present findings supported an acceptable fit of a modified four-factor model of QoL, the social domain displayed a marginal reliability, equal to what others have found [13, 14, 17, 18, 28, 37, 45–49]. A reason for the low reliability may be the low number of items [3] since the internal consistency tends to improve with increasing number of indicators [50], and thus the *true* reliability may be underestimated when the items are few [51]. Despite poor reliability of the social domain, each item had medium to strong factor loadings and explained a substantial amount of variance in the latent domain. These modifications should be considered when evaluating the overall construct validity and consistency of the instrument.

The response distributions showed that data were skewed to higher scale scores on all items and domains. Both single items and the four domains showed non-normal distributions. Such ceiling effects are well documented in QoL research [10, 14, 37], and may indicate that the range of response options is inadequate and causes poor sensitivity and responsiveness of specific items/scales [29]. However, the environmental domain is reported to discriminate sufficiently between those living in residential and those of slum areas [52], and thus the discriminatory power of the environmental domain may be better with people experiencing distinct differences in environmental resources, or with populations suffering permanent changes in their environmental well-being (i.e. in polluted areas or in physical disasters).

Results of a recent meta-analysis (24 studies, n = 2084) found evidence of small changes for the social and environmental domains and recommended investigating selected settings where, apriori, the social and environmental domains could be expected to respond significantly (positively or negatively) to types of events [15]. Importantly, one of the strengths of the WHOQOL-Bref is the inclusion of an environmental domain which often is lacking in other QoL instruments. Further work should therefore consider developing more sensitive response options for the most affected items.

In the current investigation, measurement invariance was supported for both gender and education, which findings are in line with Lin, Li [22], who reported the same results for an older Thai population.

In general, measurement invariance was supported across gender, age and education. Separate models showed a good fit for ages 18–39, but an increasingly poorer fit for age groups 40–59 and 60–75 years of age. One explanation for our findings may be that different groups may have varying linguistic interpretations of test items and category labels [30]. A differentiated subjective evaluation among older individuals are reported among a sample of older adults with post-polio syndrome [32]. Likewise, Liang and colleagues [53] found three items showing Differential Item Functioning (DIF), indicating a potential bias when using the scale in different age groups. Finally, others have noted a linear effect on the environmental domain, that is, with increasing age environmental QoL increased [2, 54]. Conceptually, it is therefore conceivable, that aged people may possess a more holistic sense of their functioning, in addition to a more differentiated subjective evaluation of specific health and QoL domains which differs from other age groups [2]. In addition, older people are to a larger degree impacted by their cultural and environmental contexts in different ways [55, 56]. Notably, over a decade ago, the WHOQOL assessment group, questioned whether other factors may be specifically important to older adults’ QoL which were not included in the WHOQOL-Bref. Consequently, an add-on module, known as the WHOQOL-Old Module, was developed and tested among 5566 older adults worldwide. Domains in this model included items related to sensory abilities, autonomy, past-present-future activities, social participation, death and dying and intimacy which have been found to be particularly important to older adults [57–60]. The results of our study may lend theoretical justification for the use of this WHOQOL-Old module together with the WHOQOL-Bref in future studies focused on older adults.

Convergent validity of the scale was shown as scale domains were found to be significantly positively correlated with overall quality of life and satisfaction with health. Furthermore, the four domains of WHOQOL-Bref were all positively correlated with each other, and work engagement. Convergent and discriminate validity of the WHOQOL-Bref has been supported in several international studies [10, 14, 15, 38].

Our normative data presesented in Table 8 are similar with the findings of Hanestad et al. [28]. In addition, we extend previous research by providing normative data for gender, different groups of age and education.

In summary, the present study has yielded updated validation data for the Norwegian WHOQOL-Bref and provided population norms. Normative data is especially useful for defining a baseline to compare the QoL in different populations. Population norms are also important to interpret Qol scores in clinical settings and to further develop and provide adequate treatments and policies. On an empirical level, it seems logical to conclude that there exist scale differences in generic Qol across cultures and that Qol is affected in a complex way by a broad array of factors [61]. Therefore, issues of invariance should not be underestimated in the performance of the scale items and domains [62]. Future studies should continue to examine measurement equivalence among various groups, especially among aged persons across different demographics. We recommend studies of individuals older than 75 years, which was the oldest age in the present study.

The results presented here cannot directly generalize to other cross-national samples. Our response rate was only 22%. The use of postal survey data makes it difficult to assess bias and reasons for non-responses [63]. Furthermore, the vast majority of participants appraised themselves as rather healthy which may explain the poor fit of the “medical treatment” and “health services” items on the domain of physical quality of life.

## Conclusions

This study suggests that the WHOQOL-Bref is suitable for use in Norway with samples from the general population. The current research supported the construct validity by providing evidence for acceptable convergent and discriminate validity and internal consistency of the physical, psychological and environmental domains, as well as invariant factor loadings across gender, education and age.

## Data Availability

The datasets used and/or analysed during the current study are available from the corresponding author on reasonable request.

## Abbreviations

QoL: Quality of life
WHOQOL-Bref: World Health Organization’s Quality of Life Questionnaire
UWES-9: The Utrecht Work Engagement Scale short version
SPSS: Statistical Package for Social Sciences
CFA: Confirmatory Factor Analysis
WLSMV: Weighted Least Square Mean and Variance adjusted
RMSEA: Root Mean Square Error of Approximation
SRMR: Standardized Root Mean Square Residual
FIML: Full Information Maximum Likelihood
MI: Modification Indices
EPC: Expected Parameter Change
DIF: Differential Item Functioning
